# Authentic Leadership and Job Embeddedness Among Clinical Nurses: Mediating Roles of Perceived Insider Status and Organisational Identification

**DOI:** 10.1002/nop2.70457

**Published:** 2026-02-15

**Authors:** Jiajia Duan, Jinping Li, Yizhou Tan, Rui Jiao, Xiangying Liu

**Affiliations:** ^1^ Department of Nursing Wuhan University of Science and Technology Wuhan Hubei China; ^2^ Institute of Nursing Research, Hubei Province Key Laboratory of Occupational Hazard Identification and Control, School of Medicine Wuhan University of Science and Technology Wuhan Hubei China

**Keywords:** authentic leadership, job embeddedness, nurses, organisational identification, perceived insider status

## Abstract

**Aim:**

This study aimed to investigate the mediating role of perceived insider status and organisational identification between authentic leadership and job embeddedness among Chinese nurses.

**Background:**

Previous studies have explored the influence of authentic leadership on job embeddedness in the nursing profession. However, the chain‐mediating effect of perceived insider status and organisational identification between authentic leadership and job embeddedness has not been clarified among nurses.

**Design:**

A cross‐sectional study.

**Methods:**

A structural equation model was utilised to examine the proposed hypothesis regarding job embeddedness in Chinese clinical nurses and to investigate potential mediating factors influencing nurses' job embeddedness.

**Results:**

There were positive correlations among authentic leadership, perceived insider status, organisational identification, and job embeddedness. Moreover, authentic leadership exerted a noteworthy influence on job embeddedness through three significant indirect pathways: the separate mediating effect of perceived insider status and organisational identification, and the chain mediating effect of perceived insider status and organisational identification.

**Conclusion:**

Nursing managers aiming to enhance nurses' job embeddedness. Recognising the crucial role of perceived insider status and organisational identification as mediators between authentic leadership and job embeddedness, our findings suggest actionable strategies. Elevating authentic leadership creates a supportive environment, positively impacting nurses' commitment and reducing turnover.

**Patient or Public Contribution:**

We would like to thank the clinical nurses from three hospitals in Wuhan who participated in the study and the hospital managers who supported this study. This study distributed survey links in the WeChat groups of three hospitals, and collected research data under the principle of ensuring anonymity and informed consent.

## 1 Introduction

The global healthcare system is experiencing an intensifying nursing shortage driven by shifting care models and rapidly aging populations (World Health Organization [WHO] 2020). The World Health Organization (WHO) estimates that the worldwide deficit of nurses may reach 6 million by 2030 (World Health Organization [WHO] 2020). High turnover amplifies this shortage and threatens workforce stability, making the identification of key factors that promote nurse retention a pressing priority. Job embeddedness, first conceptualised by Mitchell et al. (2001), offers a comprehensive explanation for why employees stay in their organisations by considering the overall forces of fit, links, and sacrifice (Al‐Ghazali 2020). In nursing, higher job embeddedness reflects strong interpersonal connections, organisational alignment, and meaningful work relationships, all of which help reduce turnover intentions (Lee and Lee 2022; Wang et al. 2025). Importantly, job embeddedness has been shown to predict retention more effectively than traditional variables such as job satisfaction or organisational commitment (Wang et al. 2024), highlighting its relevance in contemporary workforce research.

Despite its importance, empirical research examining the antecedents of job embeddedness among nurses remains relatively limited. Authentic leadership—a relational and ethical leadership style characterised by transparency, moral standards, and self‐awareness—has emerged as a potentially influential factor in shaping job embeddedness (Walumbwa et al. 2008). However, the psychological mechanisms underlying this relationship are still insufficiently understood. Specifically, evidence suggests that perceived insider status and organisational identification may independently or jointly mediate the effects of authentic leadership on retention‐related outcomes (Shahzadi et al. 2024; Dechawatanapaisal 2018). Yet, no existing research has simultaneously examined these constructs within a unified chain‐mediation framework. This gap is particularly acute in the Chinese nursing context, where organisational belonging and interpersonal trust are especially meaningful for professional stability.

To address these limitations, the present study proposes and tests a chain mediation model exploring how authentic leadership influences job embeddedness through perceived insider status and organisational identification. The central problem this study addresses is the lack of an integrated theoretical explanation for the psychological transmission mechanisms linking leadership and job embeddedness in nursing. Accordingly, this research aims to (a) assess the current level of job embeddedness among Chinese clinical nurses, (b) examine the direct effect of authentic leadership on job embeddedness, (c) test the independent mediating effects of perceived insider status and organisational identification, and (d) investigate their sequential chain‐mediating effect. Through this, the study seeks to offer theoretical insights and evidence‐based guidance for strengthening nurse retention and enhancing workforce sustainability.

## 2 Literature Review

### 2.1 Job Embeddedness in Nursing

Job embeddedness theory posits that the extent to which individuals are connected to their work environment, perceive compatibility with it, and recognise potential losses from leaving collectively functions as a stabilising force reducing turnover intentions (Mitchell et al. 2001). Substantial variation in job embeddedness is evident among nurses across different national contexts. While Egyptian nurses report relatively high embeddedness (Atalla et al. 2025) and Chinese nurses show moderate levels (Zhai et al. 2025), the United States presents a stark contrast, with nurses exhibiting significantly lower job embeddedness (Abd‐Elrhaman et al. 2020).

Existing literature has identified both organisational and individual determinants of job embeddedness. Organisational factors include organisational fit (Karadaş et al. 2025), leadership style (Wang, Hangeldiyeva, et al. 2022; Wang, Jiao, and Li 2022), and compensation systems (Song et al. 2025), while individual factors encompass self‐efficacy (Kim and Park 2023) and person–job fit (El‐Gazar et al. 2022). Positive leadership—such as humble leadership (Zhou et al. 2021) and authentic leadership (Zhou et al. 2025)—has been shown to strengthen job embeddedness, whereas stress and limited promotion opportunities impede it (Huning et al. 2020). Despite these advancements, current research frequently examines single antecedents in isolation, and comprehensive models explaining the interplay of multiple psychological pathways remain limited (Mehmood et al. 2023). These limitations underscore the need to investigate not only whether leadership influences embeddedness but also how it does so through underlying psychological mechanisms.

### 2.2 Authentic Leadership and Its Influence on Nurses

Authentic leadership, rooted in positive psychology, refers to a leadership style built on self‐awareness, relational transparency, balanced information processing, and internalised moral values (Walumbwa et al. 2017). It is considered foundational to other positive leadership styles (Walumbwa et al. 2008) and complements gaps left by traditional leadership theories (Allan and Rayan 2023). In nursing, authentic leadership is particularly relevant due to the emotionally demanding and relational nature of clinical work.

Empirical evidence suggests that authentic leadership contributes to supportive work environments that facilitate greater job satisfaction and job embeddedness (Zhou et al. 2025). Authentic leaders model ethical behaviour, demonstrate sincerity, and encourage open communication, thereby fostering harmonious interpersonal relationships (Raso et al. 2021; Batista et al. 2021). Their commitment to fairness and balanced decision‐making reinforces nurses' perceptions of organisational support (Allan and Rayan 2023), which enhances positive work attitudes and strengthens job embeddedness (Labrague et al. 2021; Zhou et al. 2021). While these findings support the direct relationship between authentic leadership and job embeddedness, emerging research suggests that indirect pathways—particularly psychological perceptions formed through social interactions—may also play meaningful roles.

### 2.3 Perceived Insider Status as a Mediator

Perceived insider status reflects employees' sense of being accepted and valued members of the organisation (Stamper and Masterson 2002). In nursing, it indicates a nurse's perceived inclusion, trust, and legitimacy within the work unit, which influence their willingness to contribute actively (Jiao et al. 2024). This construct holds particular relevance within collectivist cultural contexts like China, where organisational belonging and equitable treatment are central to professional stability (Wu et al. 2020). Research consistently links higher perceived insider status to more positive work attitudes, stronger team involvement, and improved communication (Liu et al. 2022; Jiao et al. 2024).

A growing body of research indicates that authentic leadership strengthens perceived insider status by cultivating a work environment characterised by support, transparency, and mutual respect (Li and Zhang 2022). Moreover, perceived insider status has been widely recognised as a psychological mediator linking leadership behaviours to various employee outcomes (Wang, Hangeldiyeva, et al. 2022; Wang, Jiao, and Li 2022), suggesting its potential role as an explanatory mechanism between authentic leadership and job embeddedness.

Underpinning this mediation pathway is social exchange theory (Homans 1958), which asserts that individuals tend to reciprocate beneficial treatment with favourable attitudes and behaviours. When authentic leaders demonstrate integrity, support, and transparency, nurses are more likely to perceive themselves as valued insiders and respond with stronger affective commitment and embeddedness at work (Wang, Hangeldiyeva, et al. 2022; Wang, Jiao, and Li 2022). Evidence from prior studies confirms that perceived insider status serves as a mediator in leadership–outcome relationships (Li and Zhang 2022; Wang, Hangeldiyeva, et al. 2022; Wang, Jiao, and Li 2022), thereby supporting its theoretical position as the first mediator in the proposed model.

### 2.4 Organisational Identification as a Mediator

Organisational identification refers to the extent to which individuals define themselves through their membership in the organisation (Marstand et al. 2021). Among nurses, it manifests as a sense of departmental or institutional belonging, internalised organisational values, and a stable professional identity (Haj et al. 2023). High organisational identification is consistently associated with positive psychological well‐being, improved attitudes, and greater organisational commitment (Ma et al. 2023; Jeanson and Michinov 2020). Perceptions of organisational social responsibility further strengthen identification, contributing to enhanced performance, involvement, and overall quality of work life (Jeanson and Michinov 2020; Wang et al. 2017). These positive outcomes collectively reinforce job embeddedness (Hwang and Jang 2020; Li 2021).

Research also suggests that perceived insider status may enhance organisational identification, with affective commitment functioning as an intermediary (Guo et al. 2022). Moreover, authentic leadership has been shown to strengthen organisational identification both directly and indirectly through perceived organisational support (Cheng et al. 2024). However, no study has examined perceived insider status and organisational identification together as a chain‐mediating mechanism between authentic leadership and job embeddedness.

From the perspective of social identity theory, employees who perceive congruence between their own values and organisational values tend to identify more strongly with the organisation (van Knippenberg and Hogg 2001). Authentic leadership reinforces value alignment and communicates ethical consistency, thereby supporting identification. Prior research indicates that perceived insider status may also enhance identification (Guo et al. 2022), suggesting a sequential pathway from authentic leadership to insider status to identification, consistent with the chain mediation model.

### 2.5 Summary and Research Gap

Although the individual relationships between authentic leadership, perceived insider status, organisational identification, and job embeddedness are theoretically and empirically supported, a significant gap exists in understanding their sequential interplay (Zhou et al. 2025). No prior research has empirically tested a model in which perceived insider status and organisational identification serve as chain mediators between authentic leadership and job embeddedness in a nursing sample. To address this gap, the following hypotheses are proposed:

*Authentic leadership positively predicts job embeddedness*.

*Perceived insider status mediates the relationship between authentic leadership and job embeddedness*.

*Organisational identification mediates the relationship between authentic leadership and job embeddedness*.

*Perceived insider status and organisational identification sequentially mediate the relationship between authentic leadership and job embeddedness* (i.e., *a chain mediation effect*).

## 3 Methods

This study was a cross‐sectional study conducted in three tertiary hospitals in Wuhan, Hubei Province, China from March to June 2023. This study adhered to reporting guidelines as outlined in the Reporting of Observational Studies in Epidemiology. The research was approved by the Ethics Review Committee of the Medical College of Wuhan University of Science and Technology (Ethics Approval No. 2023105) and adhered to the principles of the Declaration of Helsinki.

### 3.1 Settings and Participants

The participants were clinical nurses recruited by convenience sampling in Wuhan, China. According to the sample size requirements and estimation methods of the research on influencing factors of variables, the sample size is at least 5–10 times the number of variables, which in this study is 35, so the sample size of the survey should be 175 ~ 350, and taking into account a 10% ~ 20% missed visit rate, 420 clinical nurses were investigated, and finally, 401 were valid (The effective rate was 95.48%). The inclusion criteria comprised: (a) obtaining a nurse's practice certificate; (b) working time > 1 year; (c) informed consent and voluntary participation. The exclusion criteria included: (a) interns or refresher nurses; (b) not on the job during the investigation.

Before the study began, it was approved by the nursing departments of three hospitals. Clinical nurses from these three hospitals were chosen via convenience sampling to partake in a questionnaire survey. The survey link was subsequently distributed within WeChat groups of the aforementioned hospitals. To ensure adherence to inclusion and exclusion criteria, participants were limited to one complete submission per IP address.

### 3.2 Measures

#### 3.2.1 Job Embeddedness

Job Embeddedness Scale was developed by Crossley et al. (2007). The scale is a single dimension and contains 7 items. Likert 5‐level scoring system was used, and the scores were from 1 (“strongly disagree”) to 5 (“strongly agree”), with a total score of 7–35. As the score increases, so does the job embeddedness level of the interviewee. In this study, the Cronbach's α for this scale was 0.848.

#### 3.2.2 Authentic Leadership

Authentic Leadership Questionnaire was used. The scale was developed by Walumbwa et al. (2008). The scale mainly includes four dimensions: transparent relationship (5 items), internalised morality (4 items), balanced machining (3 items), and self‐consciousness (4 items), with a total of 16 items. Likert 5‐level scoring system was used, and the scores were from 1 (“strongly disagree”) to 5 (“strongly agree”), with a total score of 16–80. The higher the score, the better nurses perceived the authentic leadership of the leader. In this study, the Cronbach's *α* for this scale was 0.967.

#### 3.2.3 Perceived Insider Status Scale

Perceived Insider Status Scale was developed by Stamper and Masterson (2002). The scale is a single dimension and contains 6 items, including 3 forward measurement items and 3 reverse measurement items. Likert 7‐level scoring system was used, ranging from 1 (“completely disagree”) to 7 (“completely agree”), yielding a total score of 6–42 points. Higher scores indicate a greater perceived insider status. In this study, the Cronbach's *α* for this scale was 0.866.

#### 3.2.4 Organisational Identification Scale

Organisational Identification Scale was developed by Mael and Tetrick (1992). The scale is a single dimension and contains 6 items. Likert 7‐level scoring system was used, and the scores were from 1 (“completely disagree”) to 7 (“completely agree”), with a total score of 6–42 points. A greater score corresponds to an elevated perceived insider status. In this study, the Cronbach's *α* for this scale was 0.928.

#### 3.2.5 Demographic

A demographic questionnaire was compiled by researchers based on reading literature. It mainly evaluated the characteristics of participants, including gender, age, working years, education level, professional rank, work department, etc.

#### 3.2.6 Analysis

SPSS 26.0 was used for statistical analysis. The demographic characteristics of the participants and their levels of authentic leadership, perceived insider status, organisational identification, and job embeddedness were described by mean and standard deviation, frequency, and percentage. Pearson correlation analysis was used to investigate the relationship between job embeddedness and other variables. Amos 26.0 software was utilised to examine the mediating effects. The structural equation model (SEM) exploring the interplay among factors influencing job embeddedness and the causal pathways was established utilising the 5000 bootstrapped samples method with 95% bias‐corrected confidence interval (CI) estimation. A significance level of *p* < 0.05 was applied for statistical significance.

## 4 Results

### 4.1 Demographic Characteristics

A total of 401 nurses effectively participated in the study. Most nurses were female (92.0%), and 58.4% of the nurses were aged 30 or below. For educational level, 77.9% of the nurses had a bachelor's degree, and earning a master's degree or above accounted for 6.5%. For work years, approximately 46.6% had worked for less than 5 years. For professional titles, most participants had a professional title of “senior nurse” or higher level (72.8%). For work department, obstetrics and gynaecology and paediatrics account for 27.44%. For shifts, approximately 79.5% need shift work. For income, nearly half of nurses' income is less than 6000 yuan (46.2%). Moreover, 64.3% of the nurses were married. We also found that there were significant differences in the job embeddedness score among age, work years, professional rank and marital status. The specific demographic characteristics of participants are shown in Table 1.

**TABLE 1:** Characteristics of categorical variables and univariate analysis (*n* = 401).

Variable	*n* (%)	*t*/*F*	*p*
Gender			
Male	32 (7.98)	0.795	0.427
Female	369 (92.02)		
Age			
< 25	93 (23.19)	6.480	< 0.001
25–30	141 (35.17)		
31–35	89 (22.19)		
36–40	42 (10.47)		
> 40	36 (8.98)		
Education level			
College degree	90 (22.44)	2.271	0.080
Bachelor degree	285 (71.07)		
Master degree or above	26 (6.49)		
Work years			
≤ 5	187 (46.63)	3.953	< 0.001
6–10	113 (28.18)		
≥ 10	101 (25.19)		
Professional rank			
None	109 (27.17)	3.426	0.009
Junior	168 (41.90)		
Intermediate	118 (29.43)		
Senior	6 (1.50)		
Work department			
Internal medicine	109 (27.17)	1.057	0.367
Surgery	88 (21.95)		
Obstetrics and Gynaecology Paediatrics	110 (27.44)		
Other departments	94 (23.44)		
Number of shifts			
0	82 (20.45)	2.453	0.063
1–4	200 (49.87)		
5–9	90 (22.45)		
≥ 10	29 (7.23)		
Monthly income (RMB)			
< 4000	33 (8.23)	2.061	0.105
4000–6000	152 (37.91)		
6000–8000	128 (31.92)		
> 8000	88 (21.94)		
Marital status			
Single	133 (33.17)	6.147	< 0.001
Married and childless	73 (18.21)		
Married and pregnant	185 (46.13)		
Others	10 (2.49)		

### 4.2 Descriptive Statistics of Study Variables

The mean scores were 58.45 (SD = 10.99) for authentic leadership, 22.01 (SD = 4.63) for perceived insider status, 21.58 (SD = 4.54) for organisational identification, and 24.32 (SD = 4.71) for job embeddedness (Table 2). Furthermore, there were positive correlations observed among authentic leadership, perceived insider status, organisational identification, and job embeddedness. The four dimensions of authentic leadership were all positively correlated with job embeddedness. The correlations among variables are shown in Table 3.

**TABLE 2:** The mean score of authentic leadership, perceived insider status, organisational identification and job embeddedness (*n* = 401).

Variable	Score range	M	SD
Authentic leadership	16–80	58.45	10.994
Transparent relationship	5–25	18.29	3.624
Internalised morality	4–20	14.6	2.867
Balanced machining	3–15	10.93	2.155
Self‐consciousness	4–20	14.63	3.067
Organisational identification	6–42	21.58	4.544
Perceived insider status	6–42	22.01	4.634
Job embeddedness	7–35	24.32	4.706

Abbreviations: M, mean; SD, standard deviation.

**TABLE 3:** Correlation analysis of authentic leadership, perceived insider status, organisational identification, and job embeddedness (*n* = 401).

Variable	1	2	3	4	5	6	7	8
Authentic leadership	1							
2 Transparent relationship	0.946**	1						
3 Internalised morality	0.946**	0.862**	1					
4 Balanced machining	0.911**	0.802**	0.828**	1				
5 Self‐consciousness	0.943**	0.838**	0.855**	0.842**	1			
6 Perceived insider status	0.751**	0.730**	0.713**	0.652**	0.707**	1		
7 Organisational identification	0.622**	0.601**	0.583**	0.534**	0.600**	0.688**	1	
8 Job embeddedness	0.719**	0.696**	0.677**	0.615**	0.691**	0.747**	0.778**	1

** Correlation is significant at the 0.01 level.

### 4.3 Mediating Effect Analysis

**FIGURE 1:**
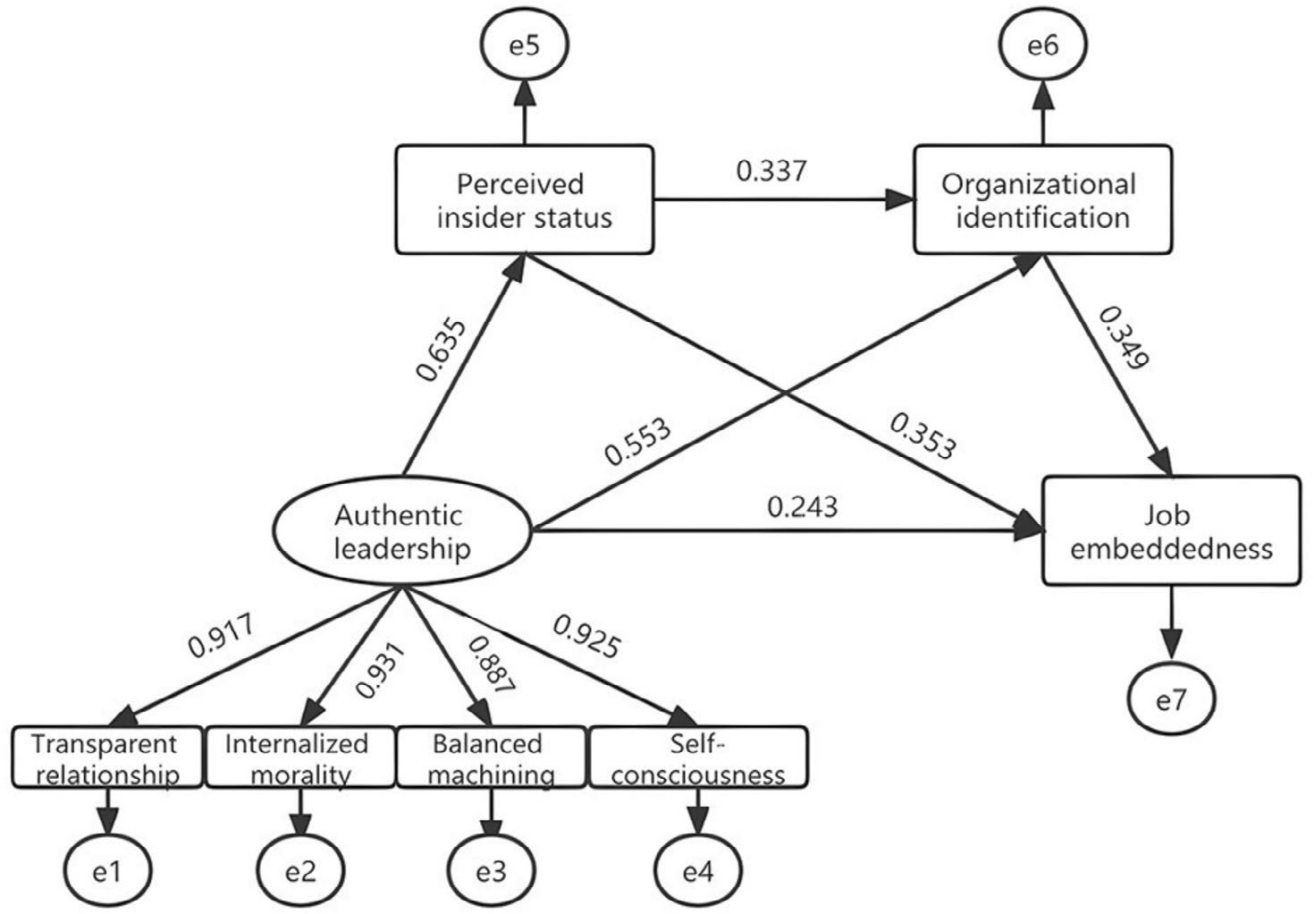
Model of the mediation role of perceived insider status and job embeddedness in the relationship between authentic leadership and job embeddedness. Standardised regression coefficients were displayed in the path diagram.

Based on the social exchange theory, social identity theory, and related literature review, as well as the correlation analysis findings, an initial structural equation model was developed to examine the mediating effect of perceived insider status and organisational identification between authentic leadership and job embeddedness, as shown in Figure 1. The result of the model fitting was as follows: *χ* ^2^/df = 2.256, RMSEA = 0.056, NFI = 0.991, IFI = 0.995, TLI = 0.991, and CFI = 0.995; therefore, the model fitted well (Table 4). The model showed that authentic leadership positively predicts perceived insider status (*β* = 0.635, *p* < 0.001) and organisational identification (*β* = 0.553, *p* < 0.001). A positive prediction exists in perceived insider status (*β* = 0.353, *p* < 0.001) and organisational identification (*β* = 0.349, *p* < 0.001) to job embeddedness; in perceived insider status (*β* = 0.337, *p* < 0.001) to organisational identification. Authentic leadership had a direct positive prediction of job embeddedness (*β* = 0.243, *p* < 0.001).

**TABLE 4:** Structural equation model fitting index.

	RMSEA	NFI	IFI	TLI	CFI
Model fitting standard	< 0.06	> 0.95	> 0.95	> 0.95	> 0.95
Model fitting index	0.056	0.991	0.995	0.991	0.995

Abbreviations: CFI, comparative fit index; IFI, incremental fit index; NFI, normal fit index; RMSEA, root‐mean‐square error of approximation; TLI: tucker‐lewis index.

The Bootstrap method was employed for model validation, running 5000 iterations with a 95% confidence interval. Findings revealed that the 95% confidence intervals for both the direct and indirect effects of authentic leadership on job embeddedness did not encompass zero, indicating statistical significance. This suggests that authentic leadership primarily influences job embeddedness through perceived insider status and organisational identification. Detailed test results can be found in Table 5.

**TABLE 5:** Effect results of the mediating model.

Path	Effect	SE	95% CI	*p*	Relative mediation effect
**Direct effect**					
Authentic leadership → Job embeddedness	0.243	0.043	0.154–0.334	< 0.001	33.06%
**Indirect effect**					
Authentic leadership → Perceived insider status → Job embeddedness	0.224	0.027	0.166–0.272	< 0.001	30.48%
Authentic leadership → Organisational identification → Job embeddedness	0.193	0.029	0.131–0.247	< 0.001	26.26%
Authentic leadership → Perceived insider status → Organisational identification → Job embeddedness	0.075	0.018	0.049–0.120	< 0.001	10.20%
Total effect	0.735	0.024	0.685–0.781	< 0.001	100%

Abbreviations: CI, confidence interval; SE, standard error.

## 5 Discussion

This study investigated Chinese clinical nurses, aiming to explore the correlation between authentic leadership and nurses' job embeddedness, to determine whether perceived insider status and organisational identification serve as mediating factors in the relationship between them, to verify all the hypotheses.

This study identified a significant positive correlation between authentic leadership and nurses' job embeddedness. That is, the higher the level of authentic leadership, the higher the level of nurses' job embeddedness. As the embodiment of the leader's overall personality, leadership style has been proven to be a crucial determinant influencing employees' attitudes and behaviours (Zhou et al. 2021). The research of Bulent et al. showed that the behaviour of high‐quality leaders provides employees with resources related to job embeddedness (Akkaya et al. 2022). This was consistent with Dechawatanapaisal's research, which found that leaders with high‐quality behaviours can influence employees' job embeddedness (Dechawatanapaisal 2018). As a highly regarded leadership style, authentic leadership creates a supportive working environment, nurturing employees' independence and passion for their work. Employees who receive support from their leaders tend to demonstrate proactive behaviour, thereby enhancing their job embeddedness (Lindsay and Mathieson 2022). American Association of Critical‐Care Nurses [AACCN] (2016) recognised authentic leadership as a fundamental element in fostering a healthy and secure work environment. The research of Allan and Rayan (2023) also found that clinical nurses who perceive authentic leadership characteristics in their leaders tend to report higher levels of job satisfaction and organisational commitment, leading to longer tenure with their employer. The viewpoint of social exchange theory also provides a framework for explaining the relationship between authentic leadership and nurses' job embeddedness. When the core characteristics of authentic leadership, such as self‐confidence, optimism, hope, and adaptability, and the virtues of promoting nurses' development, are fully displayed (Labrague et al. 2021), nurses will also improve their job embeddedness to give back to their leaders. Study Hypothesis 1 was confirmed.

This study reveals that perceived insider status significantly mediates the relationship between authentic leadership and nurses' job embeddedness. In other words, higher levels of authentic leadership correspond to elevated perceived insider status, resulting in increased job embeddedness among nurses. Authentic leadership, within the realm of leadership styles, stands out as a distinctive approach characterised by the fusion of openness and clarity (Doherty and Hunter Revell 2020; Almutairi et al. 2025). This leadership style is distinguished by its embodiment of noble moral values, the application of fair and objective decision‐making principles, and a commitment to high levels of sincerity and transparency (Raso et al. 2021). Such leadership serves as a facilitator in establishing genuine relationships with subordinates, thereby enhancing their sense of identification and trust in the leadership (Wong and Walsh 2020). Simultaneously, authentic leadership is dedicated to nurturing the strengths of subordinates, particularly emphasising their professional growth and career development. This commitment is consistently demonstrated through the display of respect and care for subordinates (Almutairi et al. 2025). As a result, subordinates under authentic leadership experience a profound acceptance and recognition of their identity within the organisational context, thereby reinforcing their perceived insider status (Jiao et al. 2024).

The study found that nurses possessing a robust sense of perceived insider status actively seek to establish connections with the organisation and its members, thereby densifying their social networks (Shahzadi et al. 2024). Moreover, they are more inclined to enhance their alignment with the internal and external environment of the organisation, which in turn increases their recognition by superiors and other members (Xu et al. 2022). The “insider” identity brings numerous resources to individuals, and leaving the organisation would entail sacrifices in material and spiritual aspects, thus leading to a higher degree of job embeddedness (Wang et al. 2021). A study on employees in Chinese enterprises showed that enhancing employees' sense of perceived insider status can improve their job satisfaction, strengthen organisational commitment (Aldabbas 2022), and enhance their job embeddedness level. The results of this study are consistent with the above findings, that is, authentic leadership influences job embeddedness through the perceived insider status. Hypothesis 2 of this study was confirmed.

In addition, organisational identification also had a significant mediating effect between authentic leadership and job embeddedness, indicating that the higher authentic leadership, the higher organisational identification and the higher level of job embeddedness. Authentic leadership is characterised by a mindset of openness and inner resilience, fuelled by a pursuit of freedom and innovation. This leadership style is marked by a robust sense of self‐awareness, self‐management, and a positive emotional state (Raso et al. 2021). According to the theory of emotional contagion, the optimistic and confident demeanour of authentic leaders can instil positive emotions in nurses, thereby fostering an increased sense of organisational identification (Wang and Zhang 2019). Authentic leaders demonstrate consistency in both their words and actions, treating nurses sincerely and embodying the core value of authenticity [48]. Nurses often generalise the authentic pursuits of their leaders into collective organisational aspirations. The organisational values espoused by authentic leadership play a vital role in alleviating nurse burnout and reinforcing their recognition of the organisation (Elkholy et al. 2020). Consequently, in contexts characterised by high levels of authentic leadership, nurses develop a strong sense of organisational identification. As nurses' organisational identification strengthens, their values align more closely with the organisation, leading to a psychological acceptance and recognition of their organisational membership. This increased organisational identification intensifies their psychological connection with the organisation, augmenting their dependence on and spontaneous elevation of their connection with the organisation (Almutairi et al. 2025). As a result, nurses' job embeddedness experiences a boost. Additionally, when nurses strongly identify with their organisation, they tend to accentuate their organisational identity, thereby reinforcing emotional attachment to the organisation. This heightened emotional attachment subtly enhances nurses' supportive attitudes and job embeddedness towards the organisation (Haj et al. 2023). In summary, this study posits that authentic leadership enhances nurses' organisational identification and belongingness by shaping the organisation's pursuit of authentic values, consequently resulting in higher levels of job embeddedness.

Finally, perceived insider status and organisational identification play a chain mediating role between authentic leadership and job embeddedness. Authentic leadership enhances the cohesion of nursing teams, fostering a more positive organisational culture and work atmosphere (Alilyyani et al. 2018). It contributes to higher work efficiency and nursing quality, elevates nurses' sense of belonging and identification with the organisation, and reduces professional burnout (Mao et al. 2022; Li et al. 2021). These factors collectively serve as advantageous elements for nurses to continue their work in their respective positions, ultimately leading to a higher level of job embeddedness.

## 6 Conclusions

This study confirms the chain mediating role of perceived insider status and organisational identification between authentic leadership and nurses' job embeddedness. These findings offer important theoretical implications by validating a dual‐theoretical mechanism grounded in social exchange theory and social identity theory. The results demonstrate how authentic leadership fosters social exchange relationships that enhance perceived insider status, which subsequently strengthens organisational identification through identity processes, collectively promoting job embeddedness. Healthcare organisations should develop authentic leadership through targeted training and implement inclusive practices to enhance nurses' insider status. Strengthening organisational identification through value alignment and shared identity building can further improve job embeddedness, offering actionable strategies to address nurse retention and stabilise workforce structure.

## 7 Limitations and Future Research Directions

This study has several limitations that should be considered when interpreting the findings. First, the cross‐sectional design restricts the ability to draw causal conclusions about the relationships among authentic leadership, perceived insider status, organisational identification, and job embeddedness. Future research employing longitudinal or experimental designs would help clarify causal pathways and capture the temporal dynamics of these variables. Second, data were collected solely through self‐report measures, which may introduce common method bias despite the procedural and statistical controls implemented in this study. Subsequent studies could incorporate multi‐source assessments or objective indicators to strengthen the robustness of the findings. Third, participants were recruited from a single city through convenience sampling, which may limit the generalisability of the results to broader or more diverse nursing populations. To enhance external validity, future studies are encouraged to replicate the investigation across multiple institutions, regions, or cultural contexts.

## Author Contributions

Jiajia Duan and Jinping Li designed the study and provided the data. Jiajia Duan and Rui Jiao conducted data analyses and prepared tables. Jiajia Duan wrote the main manuscript text, and Jinping Li supervised the study and provided valuable comments during the drafting of the manuscript. Yizhou Tan and Xiangying Liu edited the manuscript and provided valuable comments. All authors reviewed and approved the manuscript.

## Funding

The authors have nothing to report.

## Ethics Statement

The study received approval from the Ethics Review Committee of the Medical College of Wuhan University of Science and Technology (approval number: 2023105) and adhered to the principles of the Declaration of Helsinki.

## Consent

I hereby declare that I agree to the publication of the research findings presented in this manuscript in the nursing open. I have carefully read and understood the submission guidelines and requirements of the journal. I certify that the data, figures, and images presented in this manuscript are original or have been authorized and licensed legally. The subjects of this study were nurses, not patients, and informed consent has been obtained nurses.

## Conflicts of Interest

The authors declare no conflicts of interest.

## Data Availability

The authors have nothing to report.
